# Linear and non-linear combination forecasting model of varicella incidence in Chongqing

**DOI:** 10.1017/S0950268821001813

**Published:** 2021-08-02

**Authors:** Hongfang Qiu, Han Zhao, Qi Chen, Qiyin Wang, Rong Ou, Mengliang Ye

**Affiliations:** 1 Department of Epidemiology and Health Statistics, School of Public Health and Management, Chongqing Medical University, Chongqing400016, China; 2 Chongqing Municipal Center for Disease Control and Prevention, Chongqing400042, China; 3 Department of Medical Informatics Library, Chongqing Medical University, Chongqing400016, China

**Keywords:** Hybrid model, LS-SVM, SARIMA, varicella

## Abstract

Varicella is a highly infectious contagious disease, and Chongqing is one of the high incidence areas in China. To understand the epidemic regularity and predict the epidemic trend of varicella is of great significance to the risk analysis and health resource allocation in the health sector. First, we used the ‘STL’ function to decompose the incidence of varicella to understand its trend and seasonality. Second, we established SARIMA model for linear fitting, and then took the residual of the SARIMA model as the sample to fit the LS-SVM model, to explain the non-linearity of the residuals. The monthly varicella incidence peaks in April to June and October to December. Mixed model was compared to SARIMA model, the prediction error of the hybrid model was smaller, and the RMSE and MAPE values of the hybrid model were 0.7525 and 0.0647, respectively, the mixed model had a better prediction effect. Based on the study, the incidence of varicella in Chongqing has an obvious seasonal trend, and a hybrid model can also predict the incidence of varicella well. Thus, hybrid model analysis is a feasible and simple method to predict varicella in Chongqing.

## Introduction

Varicella is a highly infectious contagious disease caused by varicella-zoster virus [1]. Relevant studies have shown [2, 3] that varicella has obvious seasonality, with one or two peaks per year, often breaking out in winter and spring in temperate regions. In Spain, the incidence of varicella peaked from May to July, with a low incidence in October [4]. Giammanco *et al.* showed that varicella was one of the common childhood diseases [5]. In China, Bao *et al.* [6], Cao *et al.* [7] and Bai *et al.* [8] have described the epidemic situation of varicella in Wuhan, Wuxi and Shenyang, respectively. Their studies have shown that the incidence of varicella has obvious seasonality, and it mostly happens in student groups. According to the literature [9], a total of 3 047 715 cases of varicella were reported from 2016 to 2019, including 30 deaths in China. The annual reported incidence and mortality rates were 5505/100 000 and 0.0005/100 000, respectively. In 2018, the incidence of varicella in Chongqing ranked second in China, with a rate of 120.50/100 000, second only to Jiangsu Province. Chongqing is the largest city and economic centre in Southwest China. In 2018, the permanent resident population of Chongqing was about 31.02 million, and the proportion of children aged 0–14 is about 16.93%. To analyse the characteristics of varicella epidemic and select the appropriate prediction model to forecast the incidence of varicella in Chongqing, so as to provide an important epidemiological basis for the prevention and control of varicella in the future, is the current issue to be discussed.

For the prediction models of varicella, in foreign, Soysal *et al*. conducted a temporal trend study on the incidence of varicella in Turkey [10]. Giraldo *et al*. used an infectious disease dynamic model to conduct a preliminary study of varicella [11–13]. Lee *et al.* discussed the incidence of varicella in South Korean children [14]. In China, there are more descriptive studies on varicella [15–18]. Some scholars used the infectious disease dynamics model to predict the varicella in Changsha [19] and analysed the spatial aggregation of varicella in Jilin province [20], while others used ARIMA model [21] and grey model [22] to predict the incidence of varicella. In general, the SARIMA model can only analyse the linear information, but cannot deal with the non-liner information [23]. However, the least squares support vector machine (LS-SVM) is a kind of support vector model (SVM), which is not only suitable for small samples, but also can solve non-linear information well [24].

Considering the advantages and disadvantages of the prediction methods and the amount of research data, a single prediction model and a combined prediction model were established, respectively, based on the varicella data, and the seasonality of varicella was analysed. By comparing the prediction errors of different models, the best prediction model was selected. The best prediction model was used for short-term prediction to provide reference information for the prevention and intervention of varicella in Chongqing.

## Materials and methods

### Materials

The monthly incidence of varicella in Chongqing from January 2014 to December 2018 was studied in this paper, and the monthly incidence of varicella data is primarily gained from the Chongqing CDC.

### Methods

#### SARIMA model

Compared with the ARIMA model, the SARIMA model introduces one more seasonal effect, and the modelling process is similar to the ARIMA model. The SARIMA expression is [25]

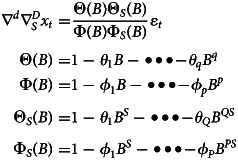


*B* is the backward shift operator, ɛ_*t*_ is the estimated residual at time *t* with zero mean and constant variance and *x*_*t*_ denotes the observed value at time *t* (*t* = 1, 2 …*k*), *s* is the length of the seasonal period, *p*, *P*, *d*, *D*, *q* and *Q* are the autoregressive order, seasonal autoregressive order, number of difference, number of seasonal difference, moving average order and seasonal moving average order, respectively.

#### SARIMA model modelling steps

First, judge the stationarity of the sequence, and make the sequence stable through appropriate methods. Second, according to the tailing and truncation of the autocorrelation coefficient and partial autocorrelation coefficient, determine the four main parameter values of the model (*p*, *q*, *P*, *Q*). Then, residual and parameter tests were carried out for the model. Compare the AIC and BIC values between the models, and choose the optimal model with the smallest two index values. Finally, the optimal model was used for prediction.

#### Hybrid model

The difference between the optimal SARIMA model-fitting value 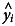 and the actual value *y*_*i*_ constitutes the residual sequence 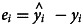, and normalise the residual sequence [26], then, fitting the LS-SVM model with the residual as the sample. Assuming a training set (*x*_*i*_, *y*_*i*_), *x* ∈ *R*, *y* ∈ *R*, *i* = 1, 2, ⋅ ⋅ ⋅ , *l*, of *l* data, *x*_*i*_ is the input data, *y*_*i*_ is the output data, and the objective optimisation function of the LS-SVM algorithm is:

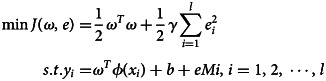

In the formula, *ϕ*( • ):*R*^*n*^ → *R*^*nh*^ is the kernel space mapping function; *e*_*i*_ is the error variable; *γ* is the adjustment parameter factor.

Sample data normalisation formula:

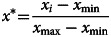


Anti-normalisation formula:

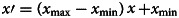

where *x*_*i*_ is sample data, *x*_max_, *x*_min_ are the maximum and minimum values of the sample data, respectively, x* is the normalised data, 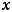 is the predicted value, *x*^′^ is the anti-normalisation value.

The root mean square error (RMSE) and mean absolute percentage error (MAPE) were used to compare the fitting effect. The RMSE and MAPE calculation formulas are [25]:

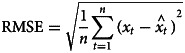


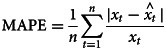


In the above equation, 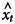 is the actual incidence value, 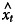 is the estimated incidence value, *n* is the amount of months for forecasting. The lower the RMSE value and MAPE value, the better the data fitting effect.

## Results

### Descriptive analyses

Table 1 shows that this study reported 112 273 varicella cases in the past 5 years (2014–2018), in Chongqing, including 58 897 males and 53 376 females, and a male-to-female ratio of 1.1034:1. Varicella mostly occurs within the ages of 0–9 years (*n* = 63 275), what is more, the age group of 0–9 accounted for 56.36% of all reported cases. The highest percentage of varicella cases was found in students, which amount to 60.74% (*n* = 68 200), followed by children in kindergarten and scattered children.

**Table 1.** Distribution of varicella by sex, age and occupation in Chongqing from 2014 to 2018

Variable	2014 *n* = 11 684 (%)	2015 *n* = 18 269 (%)	2016 *n* = 19 022 (%)	2017 *n* = 26 243 (%)	2018 *n* = 37 055 (%)
Sex					
Male	6172 (52.82)	9709 (53.14)	10 062 (52.90)	13 742 (52.36)	19 212 (51.85)
Female	5512 (47.18)	8560 (46.86)	8960 (47.10)	12 501 (47.64)	17 843 (48.15)
Age (years)					
0–9	7057 (60.40)	11 098 (60.7)	10 585 (55.65)	14 392 (54.84)	20 143 (54.36)
10–19	3283 (28.10)	5240 (28.68)	6161 (32.39)	8812 (33.58)	12 975 (35.02)
20–29	1021 (8.74)	1444 (7.90)	1626 (8.55)	2003 (7.63)	2427 (6.55)
30–39	288 (2.46)	417 (2.28)	581 (3.05)	926 (3.53)	1370 (3.70)
40–49	23 (0.20)	54 (0.30)	48 (0.25)	84 (0.32)	106 (0.29)
⩾50	12 (0.10)	16 (0.09)	21 (0.11)	26 (0.10)	34 (0.09)
Occupation					
Workers	197 (1.69)	277 (1.52)	295 (1.55)	360 (1.37)	488 (1.32)
Scattered children	1226 (10.49)	1901 (10.41)	1963 (10.32)	2455 (9.35)	3108 (8.39)
Children in kindergarten	2397 (20.52)	3842 (21.03)	3406 (17.91)	4562 (17.38)	6946 (18.75)
Students	6729 (57.59)	10 613 (58.09)	11 400 (59.93)	16 309 (62.15)	23 149 (62.47)
Farmers	227 (1.94)	339 (1.86)	354 (1.86)	453 (1.73)	612 (1.65)
Teachers	56 (0.48)	103 (0.56)	104 (0.55)	163 (0.62)	247 (0.67)
Others	852 (7.29)	1194 (6.54)	1500 (7.89)	1941 (7.40)	2505 (6.76)

### SARIMA model construction

**Fig. 1.**
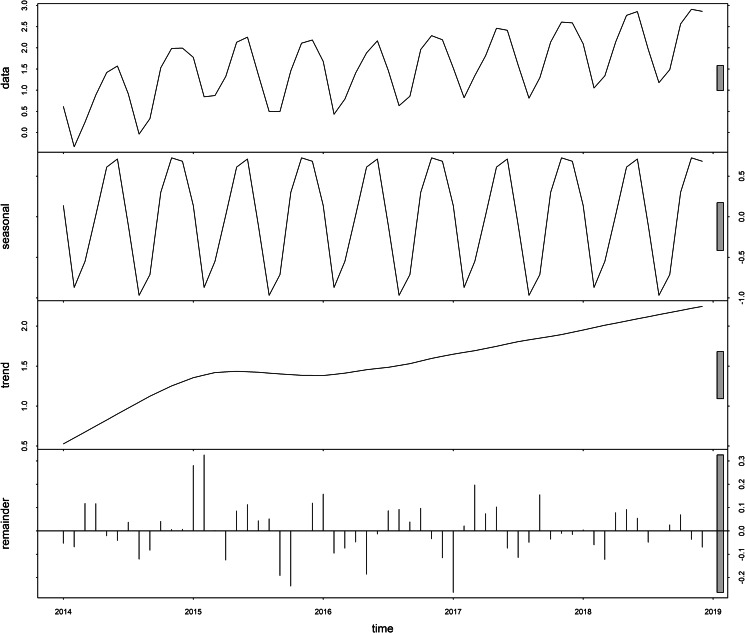
Trend, seasonal and residual components derived from ‘STL’ decomposition of monthly varicella incidence for Chongqing during 2014–2018.

**Fig. 2.**
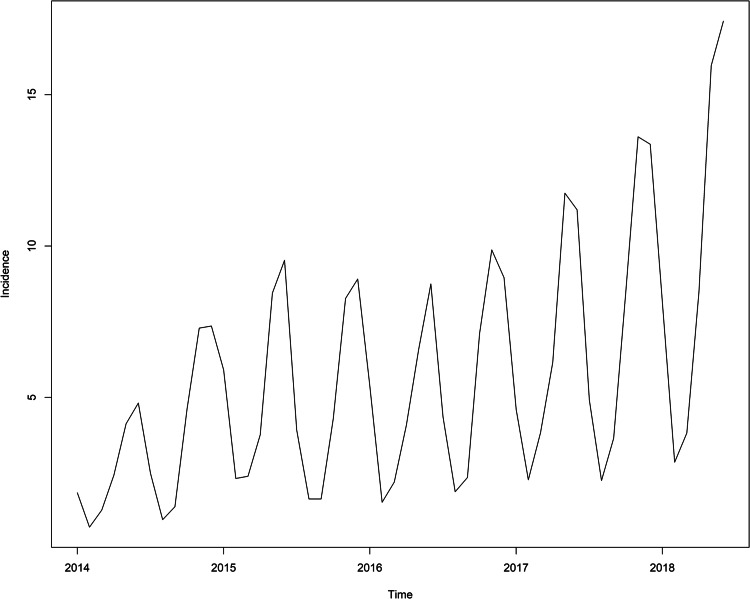
Reported monthly incidence of varicella from January 2014 to June 2018.

**Fig. 3.**
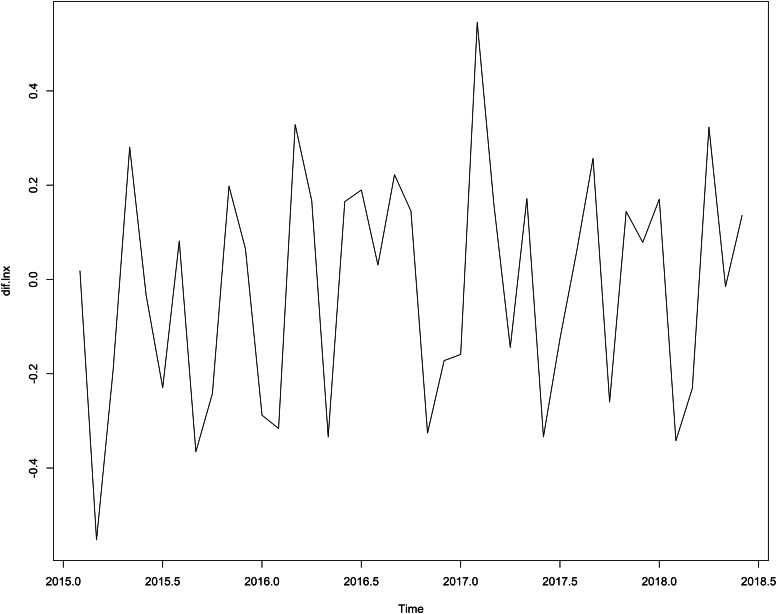
Sequence diagram after a one-step difference and seasonal difference with a period of 12.

**Fig. 4.**
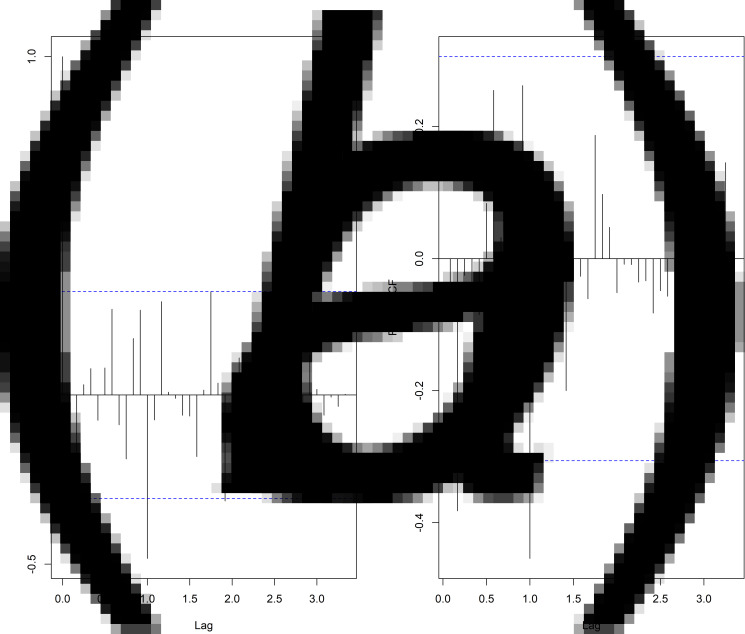
Autocorrelation function (ACF) and partial autocorrelation function (PACF) charts of monthly varicella incidence data. (a) ACF; (b) PACF.

This study used the ‘STL’ function to decompose the sequence, Figure 1 shows that the sequence has obvious seasonality, and the incidence rate presents an upward trend over time. Table 2 shows that the peak incidence of varicella was from April to June and October to December in Chongqing, and the seasonal index was >1. According to the time series diagram (Fig. 2), the monthly incidence of varicella presented a non-stationary state. After the difference processing of the original sequence, the data presented a stationary state (Fig. 3), and the unit root test showed that the sequence was stationary (*P* < 0.05). From the autocorrelation and partial autocorrelation graphs of the sequence (Fig. 4), the autocorrelation coefficient and partial autocorrelation coefficient showed tailing. Considering that the value of *p*, *q*, *P* and *Q* does not exceed 2, we verify the four parameters from 0 to 2, respectively. Only six models passed the residual test and parameter test, the six models were SARIMA(1, 1, 1) × (1, 1, 0)_12_, SARIMA(2, 1, 2) × (1, 1, 1)_12_, SARIMA(1, 1, 1) × (1, 1, 1)_12_, SARIMA(2, 1, 1) × (1, 1, 1)_12_, SARIMA(2, 1, 2) × (1, 1, 0)_12_, SARIMA(1, 1, 1) × (0, 1, 1)_12_, respectively. By comparing the AIC, BIC values and two error indicators of the six models in Table 3, SARIMA(2, 1, 1) × (1, 1, 1)_12_ model is finally selected as the best model in this paper.

**Table 2.** The seasonal index after the decomposition of ‘STL’ function

Month	January	February	March	April	May	June	July	August	September	October	November	December
Seasonal index	1.1495	0.4185	0.5781	1.0232	1.8454	2.0374	0.9039	0.3800	0.4907	1.3517	2.0686	1.9826

**Table 3.** AIC, BIC values, RMSE and MAPE for different SARIMA models

	AIC	BIC	Training set		Test set	
			RMSE	MAPE	RMSE	MAPE
SARIMA(1,1,1)(1,1,0)12	−10.19	−3.33	0.8625	0.1056	0.9804	0.0989
SARIMA(2,1,2)(1,1,1)12	−18.86	−6.86	0.5503	0.0672	1.8148	0.1186
SARIMA(1,1,1)(1,1,1)12	−14.89	−6.32	0.6744	0.0753	0.9546	0.0823
SARIMA(2,1,1)(1,1,1)12	−17.85	−7.57	0.6146	0.7422	0.7843	0.0654
SARIMA(2,1,2)(1,1,0)12	−11.29	−1.00	0.8394	0.1026	1.0455	0.0559
SARIMA(1,1,1)(0,1,1)12	−13.69	−6.84	0.7301	0.0876	1.2834	0.0753

The data on the incidence of varicella from January 2014 to June 2018 are the training set, a total of 54 data, and the data from July 2018 to December 2018 are the test set data, a total of 6.

Table 4 shows the estimated, standard errors and significance values of model parameters, all the parameter tests were statistically significant. In addition, the *P* values of LB statistics at order 6 and 12 of delay were 0.9091 and 0.6901, respectively. The white noise test of residuals was significant that indicates the fitted SARIMA(2, 1, 1) × (1, 1, 1)_12_ model was sufficient. The model equation is given as

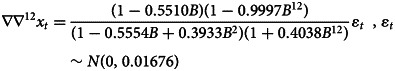

**Table 4.** Estimates and standard error of SARIMA(2, 1, 1) × (1, 1, 1)_12_ model parameters

Measurements	Model terms	Estimates	Standard error	*t* value	*P* value
Non-seasonality	AR1 term	0.5554	0.2563	2.1670	*P* < 0.05
	AR2 term	−0.3933	0.1492	2.6360	*P* < 0.05
	MA1 term	−0.5510	0.2407	2.2892	*P* < 0.05
Seasonality	Seasonality AR1	−0.4038	0.1775	2.2749	*P* < 0.05
	Seasonality MA1	−0.9997	0.4566	2.1894	*P* < 0.05

**Fig. 5.**
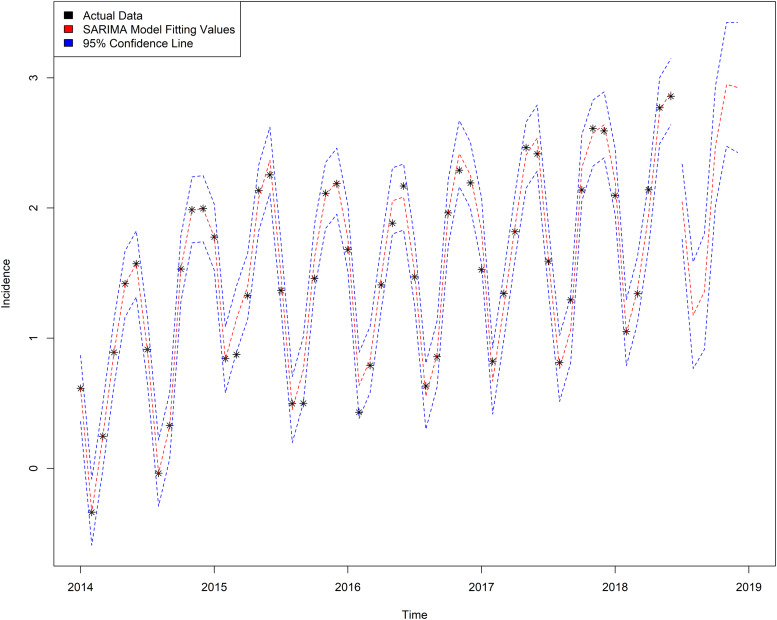
Graph of fitted and predicted values of SARIMA(2, 1, 1) × (1, 1, 1)_12_ model.

SARIMA(2, 1, 1) × (1, 1, 1)_12_ model was used to forecast the incidence of varicella. Table 5 shows the value of prediction; RMSE and MAPE values are 0.7843 and 0.0654, respectively. The actual value of incidence and fitted incidence of SARIMA model monthly is shown in Figure 5. As shown in Figure 5 and Table 5, the tendency and epidemics from predicted incidence are very close to the actual value of incidence and epidemic circumstance of varicella.

**Table 5.** Prediction of varicella incidence by two models

Time (month)	Actual incidence	SARIMA(2, 1, 1) × (1, 1, 1)_12_	Hybrid model
July 2018	7.1606	7.7538	7.6033
August 2018	3.2454	3.2415	3.2155
September 2018	4.4128	3.8887	3.7926
October 2018	13.0335	11.8913	11.6706
November 2018	18.4218	19.1106	18.7968
December 2018	17.5145	18.6482	18.4189
RMSE	–	0.7843	0.7525
MAPE	–	0.0654	0.0647

### Hybrid model construction

First, we took the residual sequence of SARIMA(2, 1, 1) × (1, 1, 1)_12_ model from January 2014 to June 2018 as the training set, the residual from July 2018 to December 2018 as the test set, and normalise the training samples. Then, we choose RBF kernel function for the LS-SVM kernel function, take different values for the embedding dimension *m* and the time delay *τ*, compare the prediction errors, and finally determine that the prediction error is the smallest when *m* is 3 and *τ* is 12. That is, using the incidence of the same period in the first 3 years to predict the incidence of the same period in the fourth year, after 50 times, iterative parameter values tend to be stable. Then, sample reconstruction was performed, and the optimal parameters *γ* and *σ* were solved by genetic algorithm with the values of 8.8540 and 110.8799, respectively, so as to establish the optimal combination model. Finally, the residual was predicted and the inverse normalisation was carried out to obtain the predicted residual value (Table 6); the predicted value of the monthly incidence of varicella obtained by the combination model was 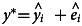 (Table 5).

**Table 6.** Residual values predicted by the LS-SVM model

Time (month)	July 2018	August 2018	September 2018	October 2018	November 2018	December 2018
Residual value	−0.1505	−0.0261	−0.0961	−0.2207	−0.3138	−0.2293

### Model comparison

**Fig. 6.**
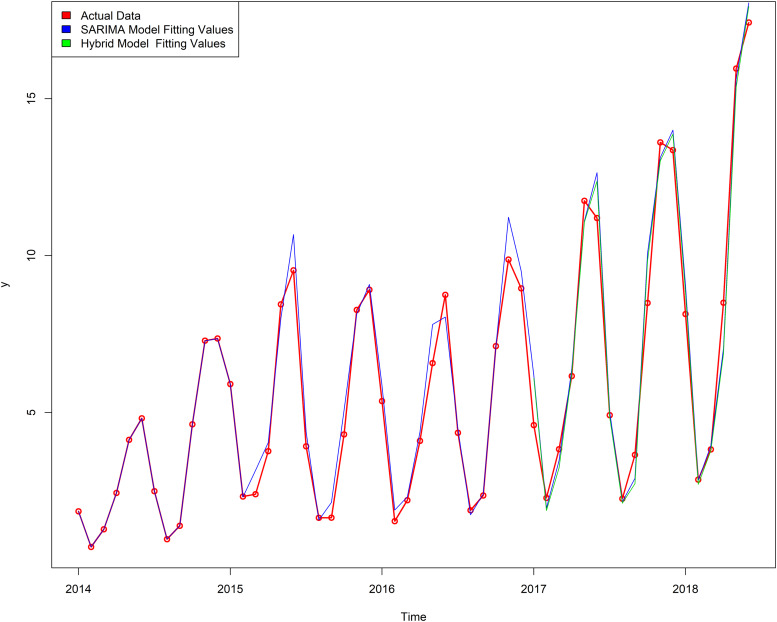
Fitting values of the two models.

**Fig. 7.**
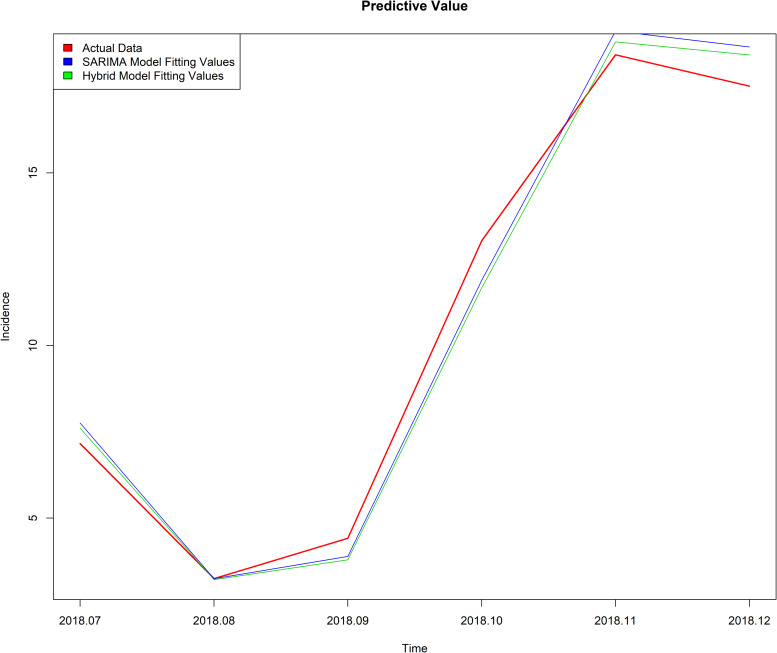
Predicted values of two models.

First, compare the fitting effects of the two models. It can be seen from Figure 6 that the fitting value of the mixed model is between the actual value and a single model. Second, a comparison of the prediction effects of the two models, from Table 5 and Figure 7, shows that the mixed model has a slightly smaller value of RMSE and MAPE, and the predicted value of the mixed model is closer to the actual value. Thus it can be seen that the best prediction model is the mixed model.

## Discussion

The descriptive analysis of varicella shows that the ratio of male and female is approximately equal, and the high incidence of varicella occurs in students, children in kindergarten and scattered children, so the incidence of varicella can be effectively controlled in this age group. The decomposing of the sequence by the ‘ STL’ function not only shows the trend and seasonal changes of the varicella incidence sequence, but also calculates the seasonal index of each month, and can intuitively understand its seasonality. In this paper, we can conclude that the peak incidence of varicella in Chongqing was from April to June and October to December, and the periods from February to March and August to September were two low stages of the disease, which is consistent with relevant studies [27–29]. The trough period may be related to the students' winter and summer vacations. During the winter and summer vacations, children's exposure opportunities are significantly reduced. Therefore, it is necessary to strengthen the intervention measures to avoid infection during the high incidence of varicella.

SARIMA model is suitable for the complex interaction among the sequential seasonal effects, long-term trends and random fluctuations. This model is one of the time series analysis models commonly used in the prediction of infectious diseases, such as tuberculosis [30], hand-foot-mouth disease [31], conjunctivitis [32], mumps [33], influenza [34] and other infectious diseases. We use the SARIMA model to perform linear fitting on the varicella series. By comparing the AIC, BIC values and combining the RMSE and MAPE values, SARIMA(2, 1, 1) × (1, 1, 1)_12_ is the best model, and the RMSE and MAPE values of this model are 0.7843 and 0.0654, respectively. It can be seen from the fitting diagram (Fig. 5) that there was a very good match between the observed values and the fitted values, the 95% CI of the forecast value contain all of the real observed data, and SARIMA(2, 1, 1) × (1, 1, 1)_12_ model can extract the deterministic information in the sequence well. Considering that infectious diseases will be affected by external factors and internal factors of the human body, with irregular changes and non-linear dynamic characteristics, the combined model of SARIMA and LS-SVM combines linear analysis with non-linear analysis.

The SVM has greater potential and better performance in practical applications [35–37]. LS-SVM uses the second square of the error as the loss function, and replaces the inequality constraints with equations to simplify the SVM algorithm, reducing the complexity of the algorithm; furthermore, LS-SVM maintains the advantages of the standard SVM. At present, SVM has been gradually introduced into the field of infectious diseases, such as bacillary dysentery [38], hepatitis B [25], hand-foot-mouth disease [39] and so on. In this study, we chose the RBF kernel function as the kernel function of the LS-SVM model; compared with other kernel functions, the parameters are easier to choose, the space complexity changed little and it was easy to implement. As can be seen from Table 5, the predicted RMSE value of SARIMA model is 0.7843 and MAPE value is 0.0654, while the predicted RMSE value of the mixed model is 0.7525 and MAPE value is 0.0647. Compared with the single SARIMA model, the mixed model has the advantage of treating the non-linear part of the residual error. In addition, Figure 6 shows that both the single model and the mixed model can well reflect the trend, peak and change trend of the actual varicella incidence. However, the fitting value and predicted value of the mixed model are between the actual value and the single model (Figs 6 and 7), indicating that the prediction effect of the mixed model is better. The mixed model can not only describe the periodicity and seasonal variation of varicella incidence in Chongqing, but also fit the non-linear part well.

In conclusion, although the prediction effect of the model is relatively good, prevention and control work should be done as soon as possible for the high incidence of varicella, strengthen daily disinfection in public places, and large-scale vaccination and other prevention and control measures should also be taken. In order to improve the accuracy of the prediction model, it is necessary to keep updating the data in the future analysis, so that the model can be optimised continuously and reflect the law and development trend of the data.

## Conclusions

Based upon the results of this study, applying the hybrid models to forecast the incidence of varicella is feasible. The fitted value and predicted value in the mixed model have the same trend as the actual value of varicella incidence, and the curve is relatively close. It suggests that a hybrid model can be used to predict the incidence of varicella. The short-term prediction of varicella is very effective, which is helpful for the evaluation of prevention or control measures. Meanwhile, we can adopt timely and effective countermeasures for the epidemic peak that may occur.

## Data Availability

The incidence of varicella data are gained from the Chongqing Center of Disease and Control; it is confidential data and cannot be uploaded to your organisation. The incidence is equal to the number of new cases of a disease in a population during a period divided by the number of people exposed during the same period.
